# Cranberry Extract and Polyphenol Metabolites Modulate Luminal and Mucosal Microbiome

**DOI:** 10.3390/ijms27198722

**Published:** 2026-09-29

**Authors:** Frank H. J. Schuren, Mirna Baak, Noemie Daniel, Bowien van Leijden, Barbara Vanhoecke, Alexandra Meynier, Margreet Heerikhuisen, Séverine Méance, Maria Wiese

**Affiliations:** 1 Microbiology and Systems Biology, TNO The Netherlands Organization for Applied Scientific Research, 2333 BE Leiden, The Netherlands; mirna.baak@tno.nl (M.B.); margreet.heerikhuisen@tno.nl (M.H.); maria.wiese@tno.nl (M.W.); 2 Health Active Solutions, Symrise, 35000 Rennes, France; noemie.daniel@symrise.com (N.D.); severine.meance@symrise.com (S.M.); 3 Global Research and Innovation, Metagenics, 8400 Oostende, Belgium; barbara.vanhoecke@metagenics.com; 4 Nutrition Research, Mondelez, 91400 Saclay, France; alexandra.meynier@mdlz.com

**Keywords:** cranberry extract, polyphenols, in vitro, mucosal and luminal microbiota, metabolites

## Abstract

Cranberry polyphenols (PPs) are associated with various health benefits, which can be exerted by the known parent compounds and/or through related metabolites resulting from microbial metabolization. Metabolite levels and profile/composition are often subject to extensive inter-individual variability, and many aspects of the relevant bidirectional interactions of PPs with the individual gut microbiota are still elusive. Both undigested and predigested cranberry extract were tested, next to procyanidin A2 and hyperoside controls, on the luminal and mucosa-associated gut microbial communities of six healthy adult donors in vitro. The composition of the microbial communities, polyphenolic substrate metabolization patterns, and microbial metabolites, including short-chain fatty acids (SCFAs), bile acids and aromatic acids, were analyzed at different timepoints. Both predigested and undigested cranberry extracts modulated the microbiota composition in all donors, albeit with individual variations. The mucosal samples responded differently than the luminal samples, and the type of extract also affected the composition of microbiota differently. Further, inter-individual differences in the metabolization patterns and metabolite production, such as 3,4 DHPPA (3,4-dihydroxyphenylpropionic acid), 3,4 DHPP-2-ol, and 3,4-dihydroxyphenyl acetic acid, were observed. SCFA production was stimulated with predigested cranberry extract. A semipolar compound analysis further showed much broader metabolic variations, both in cranberry-related metabolites, such as 4-vinylphenol and 3,4,5-trimethoxyphenylacetic acid, and in microbial metabolites such as indole-3-lactic acid. This study increases the knowledge on how individual microbiota samples are modulated upon exposure to cranberry-related PPs and how this influences their metabolic activity.

## 1. Introduction

PPs are a large group of secondary plant metabolites. Their molecular structure consists of one or more hydroxyl groups bound to one or more aromatic rings, resulting in polymers that range from simple to complex. PPs are categorized into various classes and subclasses based on their chemical structure, including the number of phenol rings and the position of functional groups [1,2]. They are found in different quantities and proportions in most plants and plant-derived beverages and form an integral part of a healthy diet [3]. Diets rich in fruits and vegetables with a substantial PP content are linked with lower disease risks [4], whereas dietary deficiencies pose a significant global health challenge in the context of rising chronic, non-communicable diseases [5]. PPs are considered nutraceuticals and have long been used in traditional Eastern medicine, Western herbal remedies, and pharmacology [6,7,8,9]. Their consumption has been associated with anticancer, antioxidant, anti-inflammatory, immunomodulatory, prebiotic-like, antimicrobial, and antiviral effects, as well as with the prevention of chronic diseases such as diabetes, obesity, neurodegenerative diseases, and cardiovascular diseases [10,11,12,13,14].

The gastrointestinal tract plays a vital role in the absorption, distribution, metabolism, and excretion of PPs. During passage through the digestive tract, secondary plant metabolites interact with other dietary components, the host, and the gut microbiota [15]. Only a small fraction (≈5–10%) of ingested PPs is absorbed in the small intestine, while higher-molecular-weight PPs usually escape absorption [16] and transit to the large intestine, where they interact with the gut microbiota in a bidirectional manner [17]. PPs may undergo microbial metabolism into lower-molecular-weight phenolic metabolites, thereby increasing their potential bioavailability [18]. Conversely, polyphenolic compounds may affect the gut microbial community by altering its structure and metabolism. The heterogeneity in response to (poly)phenol consumption is primarily associated with inter-individual differences in bioavailability [19], which largely depend on the individual’s gut microbial community structure and metabolic potential [20]. The gut microbiome harbors a collective metabolic activity comparable to that of an organ [21], and microbial genomes encode specific enzymes involved in (poly)phenol metabolization, referred to as (poly)phenol-associated enzymes (PAZymes) [22]. Because the gut microbiome composition is shaped throughout life by intrinsic and extrinsic factors such as genetic background, gender, age, medication, diet, smoking, and physical activity [23,24,25], the metabolization patterns of ingested PPs may differ substantially between individuals. Consequently, the health effects of PPs may be mediated not only by the ingested compounds and their metabolites, but also through gut microbiome-modulatory effects that influence the microbial community structure and metabolite production [26].

Cranberries are rich in polyphenolic compounds, including flavonols, anthocyanins, and oligomeric A-type proanthocyanidins [27]. Their consumption is associated with various health benefits, with the most extensively studied being beneficial effects on urinary tract health. Protective effects against cancer and vascular diseases are also being considered [28,29]. Several clinical trials have investigated the cardiometabolic effects of cranberries, indicating positive impacts on the atherosclerotic cholesterol profiles, lipid profile, endothelial function, and several markers of cardiometabolic risk [30,31,32]. Growing evidence further suggests an important role for cranberries in maintaining digestive health [33,34]. In addition to cranberry PP content, oligosaccharides may contribute to their bioactivity [35]. Therefore, the detailed biochemical characterization of fruit extracts, alongside the testing of relevant pure polyphenolic compounds, is essential to disentangle the effects of polyphenolic compounds on the gut microbiota and health [36]. Complex carbohydrates, such as arabinoxyloglucan and pectic oligosaccharides, may also play a significant role in mediating the beneficial effects of cranberries [36].

The gut microbiome-modulatory effects of cranberry extracts have been described. A recent study reported bifidogenic effects of a fully characterized, commercially available cranberry extract, Prebiocran^®^, within healthy adult microbiota. In addition to a strong bifidogenic effect, an increased abundance of several butyrate-producing bacteria, such as *Clostridium* and *Anaerobutyricum*, was reported. The authors also found that individuals with a microbiota characterized by the presence of *Prevotella* benefited from an increased *Faecalibacterium* abundance following cranberry extract supplementation [37]. Cranberry (poly)phenols have also been found to enhance the growth and abundance of *Akkermansia muciniphila*, a mucosal bacterial species associated with numerous health benefits, including potential anti-obesity effects [38,39,40]. However, it remains insufficiently clear which cranberry-derived components are primarily responsible for these microbiome-modulatory effects, how digestion alters their activity, and to what extent the responses depend on inter-individual differences in the gut microbiota composition. In particular, direct comparisons between pure cranberry-associated PPs and complex cranberry extracts, including predigested and undigested forms, remain limited. Addressing this gap is important for distinguishing compound-specific effects from matrix-dependent effects and for understanding why individuals may respond differently to cranberry-derived substrates. Therefore, this study tested the effects of pure compounds such as hyperoside and procyanidin A, as well as predigested and undigested cranberry extracts, on individual microbiotas in vitro. This approach aimed to clarify the contribution of specific cranberry PPs and cranberry extract matrices to gut microbiome modulation while circumventing the limitations of in vivo studies, such as restricted access to mucosal samples and difficulties with high-frequency sampling during digestion [41,42].

## 2. Results

### 2.1. Cranberry Extract Modulates Microbiota Composition

The effect of predigested and non-digested cranberry extracts on the luminal and mucosal microbial composition of six healthy donors (three females, three males) was analyzed in the Mi screen platform [43]. Luminal samples were collected after 6, 24, and 48 h, and mucosal samples at 48 h. Predigested extract was analyzed in two variations: E.A. dissolved in water and E.B. dissolved in 0.05% EtOH. The results are presented in Figure 1. Figure 1A shows that, although the intervention-associated shifts in the overall community structure were modest, treatment-dependent differences were detectable. Figure 1B shows the distribution of the top 50 most abundant taxa after 48 h in the luminal samples (upper panel) and the mucosal samples (lower panel). It is obvious that individual variation is the major driver of the observed differences. Moreover, clear differences between the luminal and mucosal microbiota compositions were present. *Akkermansia* was very abundant in three out of the six donors, with a higher abundance in the mucosal samples than in the luminal samples, as expected.

Significant cranberry-related effects were identified using an ANCOM BC analysis and are presented in Figure 2. This analysis shows examples of significant effects for potentially beneficial taxa that have been described in the literature to either directly respond to cranberry exposure (*Akkermansia*, *Anaerotignum*, *Bifidobacterium*, *Faecalibacterium*, *Roseburia*, *Bacteroides*, *Adlercreutzia*, *Anaerobutyricum*, *Blautia*) or to thrive in an environment created upon cranberry exposure (*Agathobacter*, *Alistipes*). Interestingly, despite the enormous individual variation in microbiome composition among the six donors, these significant effects were found in most of the donors. Comparing the data on the luminal and mucosal microbiota after 48 h of exposure (Figure 2C,D) clearly shows an overlap in the significantly affected taxa; however, differences were also observed. An analysis of samples collected at different timepoints also showed that, as of 24 h and 48 h after starting exposure, more effects were seen than after 6 h.

A direct comparison of the 48 h luminal sample composition with the 48 h mucosal sample composition showed 42 taxa that displayed significantly different abundances between the luminal and mucosal conditions (Figure 3). In the mucosal samples, a clear enrichment of the *Roseburia* species *R. intestinalis*, *R. inulinivorans* and *R. hominis* and *Eubacterium ventriosum* was seen, while under the luminal conditions, species such as *Bifidobacterium adolescentis*, *Phocaeicola dorei* and *Bilophila wadsworthia* were strongly enriched.

### 2.2. Predigestion of a Cranberry Extract Induces Slight Differences in Microbiome Composition Compared to Non-Digested Extract

Since it is not clear how small intestinal passage influences the effect of cranberry extract on the microbial composition, both undigested and digested extracts were tested for their effect on the microbiome. Since cranberry extract contains various compounds with different properties such as solubility and bio-activity, the extracts were evaluated in two different solvents (water and 0.02% ethanol). The following product variations were tested and compared:

- Undigested product directly added to microbiota;
- Predigested product in MQ (A);
- Predigested product in 0.02% EtOH (B).

Figure 4 shows the results of the ANCOM-BC analysis obtained with these three products in six different/individual fecal microbiotas cultured in vitro. Panels A,B show the comparison of all intervention conditions after 6 and 48 h. Panels C–F show the comparison of the three cranberry extract variations at different timepoints. Again, strong overlap in the microbial community composition was seen between the different exposures, with the predigested ethanol extract showing the most deviation after 6 and 24 h, and the undigested extract showing the most deviation in the luminal microbiome after 48 h. Despite the strong overall overlap in the effects, a more detailed analysis showed a number of taxa with significantly different abundances among the three extracts (Panel G). This one-to-one comparison of the extracts suggests more significant taxa in the predigested versus non-predigested comparisons versus the comparison of predigested extracts in different solvents. These effects are difficult to quantify and individual variation in the response was clearly observed. These data show that the predigestion of the product affects the microbiota composition, despite being dominated by inter-individual variation.

### 2.3. Human Microbiota Metabolizes Cranberry PPs

Next, the metabolic profile of major components of cranberry extract over time was analyzed. Next to cranberry extract, two PPs, hyperoside and procyanidin A2, were included as pure ingredients. The detected metabolites after 0, 6, 24, and 48 h are shown in Figure 5. The t = 0 samples allowed the predigestion profile to be measured, and they clearly showed the presence of myricetin in the undigested cranberry samples, but not in the predigested samples. The metabolic pattern after 6 h of incubation was clearly different from the predigestion profile, indicating that metabolization starts quickly. The parent flavonols quercetin, myricetin and hyperoside were all affected, but differentially. Quercetin and myricetin were observed in donors F1 and M3, but were absent in donors F2 and F3, with M1 and M2 showing intermediate levels. Hyperoside and coumaric acid were already completely metabolized by all donors. The parent flavan-3-ol procyanidin A2 was still detected in all samples after 6 h, but its metabolites 3,4 DHPP 2 ol, 3,4 DHPVL, 3-HPVA, and 3,4 DHPPA were also all observed after 6 h. The patterns clearly changed after 24 h, with all parent molecules now degraded. Metabolites of the parent molecules, such as 3,4 DHPP 2 ol, 3,4 DHPVL, 3-HPVA, and 3,4 DHPPA, disappeared again in some donors, indicating further metabolism. After 48 h, more of these metabolites disappeared, but not in all cases. There was a clear donor effect showing that individual variations in microbiome composition strongly influence these metabolic processes. This resembles potential metabotypes [26,44], which were shown to highly or slowly convert epicatechin into 3-HHP-2ol and 3,4-DHPVL, dependent on the microbiome composition. Figure 5 shows low production of valerolactone and phenylacetic acids in donor F2, indicative of metabotype 1, whereas high levels were seen in donors F1 and F3, indicative of metabotype 3. For donors M1, M2 and M3, the results were less conclusive. These results clearly show metabolic activity in all microbiome donor samples, which supports the hypothesis that these changes are driven by cranberry extract (related) compound metabolism.

### 2.4. Cranberry PPs Increase SCFA Acid Production by Human Microbiota

After showing that cranberry extract leads to changes in microbiome composition, including changes in beneficial taxa, which occur concomitant with the metabolism of specific cranberry compounds, the question arises as to whether this also causes the microbiome to produce specific health-related metabolites. The SCFA production was therefore analyzed in response to cranberry extract exposure. Figure 6 presents the log2 fold changes in the SCFA levels relative to the untreated control after 24 h and 48 h of exposure. All conditions in which the measured concentrations were higher compared to the untreated control condition are boxed.

After 24 h, this was the case for many of the experimental conditions (upper panel), with undigested cranberry extract and procyanidin A2 showing less stimulation than the other conditions. After 48 h (lower panel), only a limited number of conditions still showed increased production compared to the untreated control condition. As was the case for the microbiota composition effects and cranberry compound metabolism effects, individual variation between microbiota was clearly observed, with some microbiota showing a stronger response than others. As an example, the M3 microbiota seemed to be a “late responder” compared to the others. No significant differences were observed when comparing predigested and undigested extracts.

### 2.5. Cranberry PPs Modulate Human Gut Metabolome

Apart from SCFAs, there are many more metabolites that are produced by the gut microbiome. These can be both endogenously produced metabolites as well as metabolites of exogenously supplied compounds such as nutrients (e.g., amino acids). A subset of samples collected during the here-described experiments was subjected to a semipolar compound analysis, which targets compounds of intermediate polarity. The results are presented in Figure 7, which shows significant differences in the detection of selected compounds among experimental conditions.

The semipolar compound analysis allowed us to detect several other categories of bacteria-producing metabolites following the administration of cranberry extracts. Among them, a rather homogenous increase in 4-vinylphenol in the donors’ microbiota was observed, regardless of the predigestion step (Figure 7A). Likely derived from cranberry p-coumaric acid decarboxylation, this microbial phenolic catabolite may serve as a marker of the microbial transformation of cranberry phenolics or related hydroxycinnamate-like substrates. On the other hand, some other compounds such as 3,4,5-trimethoxyphenylacetic acid (a probable aromatic catabolite) displayed a more heterogenous production, depending on and significantly increased in half of the samples (Figure 7A). Some impact related to the predigestion step was also observed, such as for the asymmetric dimethylarginine, which tended to increase in the presence of the predigested extracts compared to the undigested extract. Overall, it seems that the metabolome appeared more affected by MQ predigestion than by ethanol predigestion.

The cranberry extract did not seem to greatly affect the indole/tryptamine subset of metabolites, except for indole-3-lactic acid (ILA), which was non-significantly increased under most of the conditions, suggesting that cranberry PPs favor the microbial conversion of tryptophan into health-related metabolites such as ILA with anti-inflammatory and immune-regulating properties.

Finally, a great impact of the cranberry extracts on bile acids was observed. The taurocholic acid levels were notably increased following the addition of the raw extract and the predigested extract with MQ (intriguingly, not with the ethanolic predigested extract). This indicates the capacity of the cranberry extract to modulate the microbiota–bile acid axis, potentially via reduced bacteria deconjugation or a shift in bile salt hydrolase (BSH)-active taxa.

## 3. Discussion

Cranberries are generally considered to be healthy for humans based on their high levels of PPs, antioxidants, vitamins, and fibers. In this study, the effects of cranberry extracts on the gut microbiome were observed. Based on the results presented, it can be concluded that cranberry extract does influence the microbiota composition, that the metabolization of major components in cranberry extract does occur upon exposure to the microbiome, and that the microbiome responds to this exposure by producing SCFAs and other metabolites. These results therefore further support their considered health effects. However, it is also clear that these effects are not always uniform: variations in extract preparation led to differential effects on the microbiome composition and microbial metabolite production and profiles. This study also aimed to increase our knowledge on specific cranberry extract-induced effects on different individual microbiota and their metabolization in specific microbial environments. This may help us to understand the microbiome-driven variability in response to these extracts, which is often observed and associated with differences in individual responses.

The variation in microbiome composition is best observed in Figure 1. The effect of the different extracts on the microbiome composition was inferior compared to the differences among donors. This enormous variation among donors also complicated the identification of more generic microbiome effects in the general population. Patterns in the donor response were therefore identified, which might support healthy microbiome compositions in the general population. In Figure 2, specific beneficial microbial taxa are shown that responded to the cranberry extracts in multiple donors. These include taxa previously described to respond to cranberry exposure, belonging to *Bacteroidaceae*, *Akkermansia*, *Bifidobacterium*, *Alistipes* and *Faecalibacterium* [37,38,45]. Most of these taxa have been linked to potential health effects in humans, so the stimulation of these taxa can be considered a potentially beneficial effect. In this study, the mucosal and luminal microbiome compositions were also analyzed and compared. Figure 3 clearly shows the differences between mucosal- and luminal-enriched taxa, both under control conditions and upon exposure to cranberry extracts, with multiple *Roseburia* species (*R. inulinivorans*, *R. intestinalis* and *R. hominis*) highly enriched in mucosal samples, whereas *Bifidobacterium adolescentis*, *Phocaeicola dorei* and *Bilophila wadsworthia* were highly enriched in the luminal samples. These differences between the mucosal and luminal microbiota may also have an effect on gut functionality. The mucosal microbiota is in close contact with the gut epithelial tissue and may influence barrier function and bioavailability. Future research into host–microbe interactions and the effect of PPs on these interactions could help to increase our understanding. Despite these clear differences between the mucosal and luminal microbiota, Figure 1A shows that individual variation is still the main driver of variation both in the mucosal and luminal microbiota.

With respect to cranberry extract formulation, the effect of cranberry extract not subjected to small intestinal predigestion was compared with extracts predigested according to a standardized INFOGEST protocol followed by either MQ or EtOH solubilization. Although differences in microbiome effects were observed among these extracts (Figure 4), it was also clear that all effects were partially overlapping, showing that slight variations in specific compound concentrations were probably introduced by the different extraction methods. No clear differences in health outcomes for the general population are expected based on these results. However, when aiming at targeting specific taxa in the microbiome, it certainly would be worthwhile to carefully consider different extraction procedures in order to identify an optimal formulation/composition.

To support the hypothesis that the observed changes in microbial taxa were actually related to cranberry extract exposure, the metabolism of major cranberry extract components was also analyzed. Figure 5 clearly shows the fast metabolism of these components, with major changes already seen after 6 h, and continuing after 24 h and 48 h. These data clearly show metabolic activity in the microbiome samples, but also show clear donor variations in metabolic profiles.

Besides promoting health-associated beneficial taxa, the production of microbiome-derived metabolites by the cranberry extract was also assessed, as functional readouts may provide additional insight into the potential physiological relevance of microbiome compositional changes. Figure 6 shows the results for SCFA production, with a clear stimulation of multiple SCFAs, including acetate, propionate and butyrate, in multiple donors, especially after 24 h of exposure. Since these SCFAs are involved in many different processes in the human body, this result can be considered a potential health effect. Next to SCFAs, a more diverse set of compounds (here called semipolar compounds) was also investigated. Figure 7 shows the results for semipolar compounds, which showed significant differences compared with the control samples. These include cranberry-derived metabolites such as 3,4,5 trimethoxyphenylacetic acid, homovanillic acid and 4 vinylphenol, which are probably metabolized by the microbiota. Strikingly, these metabolites were only found in some donors and not in others, with the 3,4,5 trimethoxyphenylacetic acid pattern differing from the homovanillic acid and 4 vinylphenol patterns. These results once more show the impact of inter-individual variation in microbiomes on the effect of the cranberry extract, not only on the microbiome composition, but also on the levels and profile of the derived metabolites. Other interesting metabolites that were induced in some donors were the indoles indole-3-lactic acid (ILA) and indole-3-acetic acid (IAA). ILA is a microbial metabolite with anti-inflammatory and immune-regulating properties. IAA can be a plant-derived hormone, but can also be produced by the microbiome and it has anti-inflammatory properties. These results suggest potential links between cranberry consumption and specific human health effects, mediated by the gut microbiome.

Tosi et al. recently published a study examining circulating (poly)phenol metabolites, which may act as mediators of cranberry-consumption-related health effects [26]. They identified three urinary metabotypes based on colonic phenolic metabolites, 5-(3′,4′-dihydroxyphenyl)-γ-valerolactones, which are relevant to metabotyping. The metabotype allocation was well preserved after a 12-week cranberry intervention [26]. The detection of metabotypes across studies involving different individuals can be relevant for improving our understanding of the role of gut microbiota PP interactions for health effects and personalized nutrition approaches [26]. In vitro systems may be useful in supporting such personalized approaches. Recent research showed that the effect of a single fiber dose in vitro was predictive of the effect after 8–12 weeks in humans [46]. The application of a comparable strategy for PP intervention may help to identify optimal product microbiome fits and thereby improve intervention effects in humans.

More complex interactions between the gut microbiota, PPs and host factors such as bile acids may play a role in the exertion of health effects [47,48]. Our data also show effects on bile acid metabolites. The bile acid–gut microbiota axis has been described as a central hub for physiological regulation and a novel therapeutic target for metabolic diseases [49]. By adding PPs into the mix, the PP–bile acid microbiota axis is a dynamic three-way interaction worth exploring [48], since PPs may modulate the gut microbiota and affect bile acid transformation (e.g., deconjugation, dehydroxylation). On the other hand, bile acids influence the gut microbiota composition and impact PP metabolism, which may influence the host metabolism and health status [50,51]. Taken together, this study adds to the increasing knowledge on the complexity of microbiome–PP interactions and inter-individual responses on cranberry extract supplementation, which will help design better or more targeted natural products in promoting multiple health outcomes.

## 4. Materials and Methods

### 4.1. Workflow In Vitro Screening of the Substrate Effects

An overview of the experimental setup is displayed in Figure 8.

### 4.2. Test Substrate

We investigated the effects of the following substrates: undigested cranberry extract (60 μM Cat. Eq. in MQ), predigested cranberry extract (60 μM Cat. Eq. in MQ), predigested cranberry extract (60 μM Cat. Eq. in 0.02% EtOH), an untreated control (MQ), an EtOH control (0.5%), hyperoside (49 μM Cat. Eq. in 0.5% EtOH), and procyanidin A2 (40 μM in 0.5% EtOH).

Considering that our complex cranberry PP extract was composed primarily of A-type procyanidins made up of catechin units, our approach was to adjust the compound quantities of our extract, hyperoside and procyanidin A2 using the Folin method’s phenolic response coefficients [52] to ensure the delivery of equivalent amounts of the phenolic groups, expressed as catechin equivalents. Considering a 2 L dietary bolus and the purity of the compounds, this resulted in 46.12 mg/j of procyanidin A2, 45.4 mg/j of hyperoside and 97.4 mg/j of cranberry extract for 35.04 mg/j of catechin equivalents. The concentrations selected in this way are of the same order of magnitude as those consumed in the daily food bolus [53]. The cranberry extract composition is shown in Supplementary Table S1. Hyperoside and procyanidin A2 were provided by Extrasynthese (Genay France).

### 4.3. Predigestion of the Cranberry Extract

The in vitro digestion was carried out according to the protocol published by the COST INFOGEST network [54,55]. In brief, simulated salivary fluid (SSF) with an enzymatic activity of 75 U/mL for amylase (A1031, Sigma-Aldrich Chemie B.V., Zwijndrecht, The Netherlands) was mixed with cranberry extract in a ratio of meal:SSF of 50:50 and set to a pH of 7.0 to mimic the oral phase. After 2 min of incubation at 37 °C, the solution was brought to a pH of 3.0 and subsequently mixed with simulated gastric fluid (SGF) with an enzymatic activity of 2000 U/mL for pepsin and 60 U/mL lipase (derived from rabbit gastric extract (RGE15, Lipolytech, Marseille, France)) in a ratio of meal + SSF:SGF of 50:50 and set to a pH of 3.0 to mimic the gastric phase. After 1 h of incubation at 37 °C, the solution was brought to a pH of 7.0 and subsequently mixed with simulated intestinal fluid (SIF) in a ratio of meal + SSF + SGF:SIF of 50:50 with an enzymatic activity of 100 U/mL for pancreatin (P3292, Sigma-Aldrich Chemie B.V.) and 10 mM bile salts (bovine bile B3883, Sigma-Aldrich Chemie B.V.) to mimic the intestinal phase. The solution was adjusted to a pH of 7.0, incubated at 37 °C for 1 h, and then placed at 100 °C for 5 min to inactivate the enzymes before storage at −20 °C until use.

### 4.4. In Vitro Study in the Mucosal I-Screen (Mi-Screen) Model

For the culturing of putative mucosa-associated bacteria, the 24 deep-well plates were coated with 230 μL of a mucus agar mix, which was prepared with mucin from porcine stomach type III (Sigma Aldrich) and agar (Oxoid, Haarlem, The Netherlands) for the experimental approach of a mucosal i-screen (Mi-screen) similar to that described in [43]. The polyphenolic test substrates were screened with six gut microbial communities derived from three males (aged 22, 38 and 64) and three females (aged 56, 61 and 62). All donors complied with the following criteria: an age under 65, good health, non-smoking, no GI complaints in the 2 weeks prior to donation, no drug use in the two weeks prior to donation (with the exception of vitamin pills), no antibiotic use up to 3 months prior to donation and not being on their period during donation. Before starting the Mi-screen incubations, the fecal samples of the three male and three female donors were pre-cultured for 4 h in standardized ileum effluent medium (SIEM) under anaerobic conditions, at 37 °C and with shaking at 300 rpm [56]. The microbiota was then transferred to the 24 deep-well plates for incubation in SIEM medium with the pH adjusted to 5.8 to test the influence of the test substrates on the growth of the complex gut microbial community next to the untreated controls. The inoculated plates were then covered with gas-permeable seals and incubated at 37 °C at 300 rpm under anaerobic conditions (Don Whitley Anaerobic chamber) for 48 h. Each of the experimental conditions was studied in triplicate. After 0, 6, 24, and 48 h of incubation, a sample of 150 μL was transferred into a fresh deep-well plate for gDNA extraction and subsequent molecular analyses. For the PP analysis, SCFA analysis and semipolar compound analysis, sample material was harvested and centrifuged for 10 min at 3000 rpm at 4 °C. The supernatant was centrifuged once more for 10 min at 3000 rpm at 4 °C and was split into three parts for further processing for the different types of analyses. After 48 h, all of the liquid culture was removed from the Mi-screen experimental set-up (from here onwards, the liquid culture derived from the Mi-screen is referred to as a luminal sample). Subsequently, the mucus agar layer was washed three times with 1 mL of phosphate-buffered saline (P2272, Sigma-Aldrich) before proceeding with gDNA extraction from the mucus agar layer (mucosal sample).

### 4.5. Metagenome Sequence Analysis

Genomic DNA extraction, library preparation and sequencing were performed as earlier described [43]. The sequencing data were deposited in the ENA database under accession number PRJEB123876. The sequencing reads were host (human)-filtered and quality-controlled by KneadData (v0.10.0). Taxonomic classification was performed with Metaphlan (v4.2.2) at the species level using the ChocoPhlAn database mpa_vJan25_CHOCOPhlAnSGB_202503 and the method rel_ab_w_read_stats. Taxonomic count tables and sample meta information were converted to a single phyloseq object using phyloseq (v1.54.0).

A statistical analysis was performed with R (v4.5.1) and visualization was achieved with ggplot2 (v4.0.2).

The alpha diversity indices Shannon and Choa1 were calculated with vegan (v.2.7-2). The statistical significance was assessed using dunn_test of rstatix (v0.7.3). A principal coordinate analysis (PCoA) was performed on a Bray–Curtis distance matrix calculated from sample-size-normalized counts and the relative abundance, using pcoa of ape (v5.8-1). A distance-based redundancy analysis (dbRDA) was performed using the vegan package (v2.7-2) on Bray–Curtis dissimilarities derived from Hellinger-transformed microbial abundance data. Compound effects were assessed while controlling for microbiota donor effects.

Differentially abundant taxa were identified using ANCOM-BC2 (v2.12.1) for each microbiota donor at each timepoint. The analyses were performed using default parameters with the neg_lb and pairwise options enabled and the compound specified as the fixed effect. Differences between the mucosal and luminal microbial communities were assessed across all microbiota donors, with the microbiota donor included as a covariate to account for donor-specific effects.

### 4.6. PP Analysis

One hundred microliters of the supernatant samples were transferred to deep-well plates and diluted five times in acetonitrile (ACN) containing 2 ug/mL kaempferol as an internal standard. After centrifugation for 10 min at 3000 rpm at 4 °C, the samples were transferred to autosampler plates and were stored at −80 °C until the LC-MS analysis was performed.

### 4.7. LC-MS Analysis

Samples from the various treatments were analyzed by UPLC-MS using the UPLC of Waters Acquity H-Class and the MS of Thermo Q-Exactive equipped with HESI-Source. A 20-min-long generic gradient separation (low to high organic content with some formic acid) was applied for the first samples. Here, a rather polar stationary phase was used, as it was expected that at least several of the available PPs would “per definition” be metabolized, and generally, this implies an increased polarity of the molecules. The first experiments showed that several compounds were eluted from the column during the washing stage. Consequently, the method was adapted to a steeper gradient, which resulted in faster elution. At the same time, the flow rate was increased from 0.4 to 0.6 mL/min, which increased the throughput considerably. The following equipment and conditions were used: UPLC (Waters Acquity H-Class) consisting of a QSM (quarternary pump), FTN (autosampler) and CM (column heater), with a column (Waters Acquity HSS T3 100 mm × 2.1 mm, 1.7 µm dp), a column temperature of 40 °C, mobile phase 1 of 0.1% formic in water (MilliQ, Millipore, Amsterdam, The Netherlands), mobile phase 2 of 0.1% formic acid in acetonitrile (LC-MS grade, Biosolve, Eindhoven, The Netherlands), a wash solvent of 90/10 (*v*/*v*) MeOH/MilliQ, a purge solvent of 10/90 (*v*/*v*) ACN:MilliQ, a flow rate of 0.60 mL/min, an injection chamber temperature of 10 °C, and an injection volume of 2 uL.

#### Data Processing LC-MS

MS and MS/MS data were processed using the software program “Compound Discoverer” from Thermo Scientific. The LC-MS data were processed by integrating the [M-H]^−^ ion and, in some cases, the [M + H]^+^ ions, and adducts or source-related fragments were identified through the CD program. The mass tolerance for constructing the chromatograms was 10 mmu (0.005 Da). The peak areas were divided by the peak area of the internal standard to obtain the response relative to the internal standard. The average of each sample (*n* = 2 or 3) was calculated. The data of the timepoints of each sample were visualized against the proper controls and trendlines were constructed.

### 4.8. Semipolar Compound Analysis

An SCFA analysis was performed as earlier described [43]. For the semipolar compound analysis, the supernatant from the centrifuged samples was filtered with GE Healthcare Unifilter microplate devices to remove all solid cell matter from the supernatant. In brief, after sample loading, filter plates with the samples were centrifuged for 10 min at 3000 rpm at 4 °C. The remaining cell-free supernatant was stored at −80 °C until shipped to Cmbio in Denmark for the semipolar compound analysis.

#### Sample Preparation

Microbial fermentation samples contained substantial concentrations of media-derived detergents that required removal prior to the mass spectrometry analysis. The detergents were precipitated by complexation with an aqueous solution of zinc nitrate (0.26 M) and potassium thiocyanate (2.6 M) containing stable isotope-labeled internal standards. The samples were mixed with the precipitation solution at a ratio of 3:1 (*v*/*v*), followed by centrifugation at 3200× *g* for 20 min at 4 °C. The resulting supernatants were filtered through 0.2 μm polyvinylidene fluoride filter plates. The filtrates were subsequently diluted 11-fold in a mixture of mobile phases A and B (9:1, *v*/*v*), where mobile phase A consisted of 10 mM ammonium formate and 0.1% formic acid in ultrapure water and mobile phase B consisted of 10 mM ammonium formate and 0.1% formic acid in methanol. Stable isotope-labeled internal standards were added prior to the LC-MS analysis.

### 4.9. Semipolar Metabolite Analysis

The sample analysis was carried out by Cmbio (Vedbæk, Denmark) as follows. The analysis was carried out using a Thermo Scientific Vanquish LC coupled to an Orbitrap Exploris 240 MS, Thermo Fisher Scientific, Waltham, MA, USA. The UPLC was performed using an adapted version of the protocol in [57] on a Waters Acquity Premier HSS T3 (2.1 × 150 mm, 1.7 μm) column. An electrospray ionization interface was used as an ionization source. The analysis was performed in positive and negative ionization mode under polarity switching. Fragmentation spectra were recorded in iterative exclusion-data-dependent acquisition on pooled QC samples.

#### Data Processing Semipolar Metabolites

Metabolomics processing was performed untargeted using Compound Discoverer 3.4 (Thermo Scientific) and Skyline 24.2 (MacCoss Lab Software) for peak picking and feature grouping. Further data processing, quality control, and curation were performed using custom workflows implemented in MATLAB R2024b (MathWorks, Natick, MA, USA) and R version 4.3.0.

The annotation of compounds was performed at four levels: Level 1, identification by retention times (compared against in-house authentic standards), accurate mass (with an accepted deviation of 3 ppm), and MS/MS spectra; Level 2a, identification by retention times (compared against in-house authentic standards) and accurate mass (with an accepted deviation of 3 ppm); Level 2b, identification by accurate mass (with an accepted deviation of 3 ppm) and MS/MS spectra; and Level 3, identification by accurate mass alone (with an accepted deviation of 3 ppm).

### 4.10. Data Analysis

The statistical analysis was performed with R (v4.5.1) and visualization was achieved with ggplot2 (v4.0.2). Semipolar metabolite data were log-transformed after the addition of a pseudocount 1 × 10^−9^ and centered, normalized relative to the untreated control within each condition. The statistical significance was evaluated using Student’s *t*-test with the Benjamini–Hochberg correction for multiple testing.

## Figures and Tables

**Figure 1:**
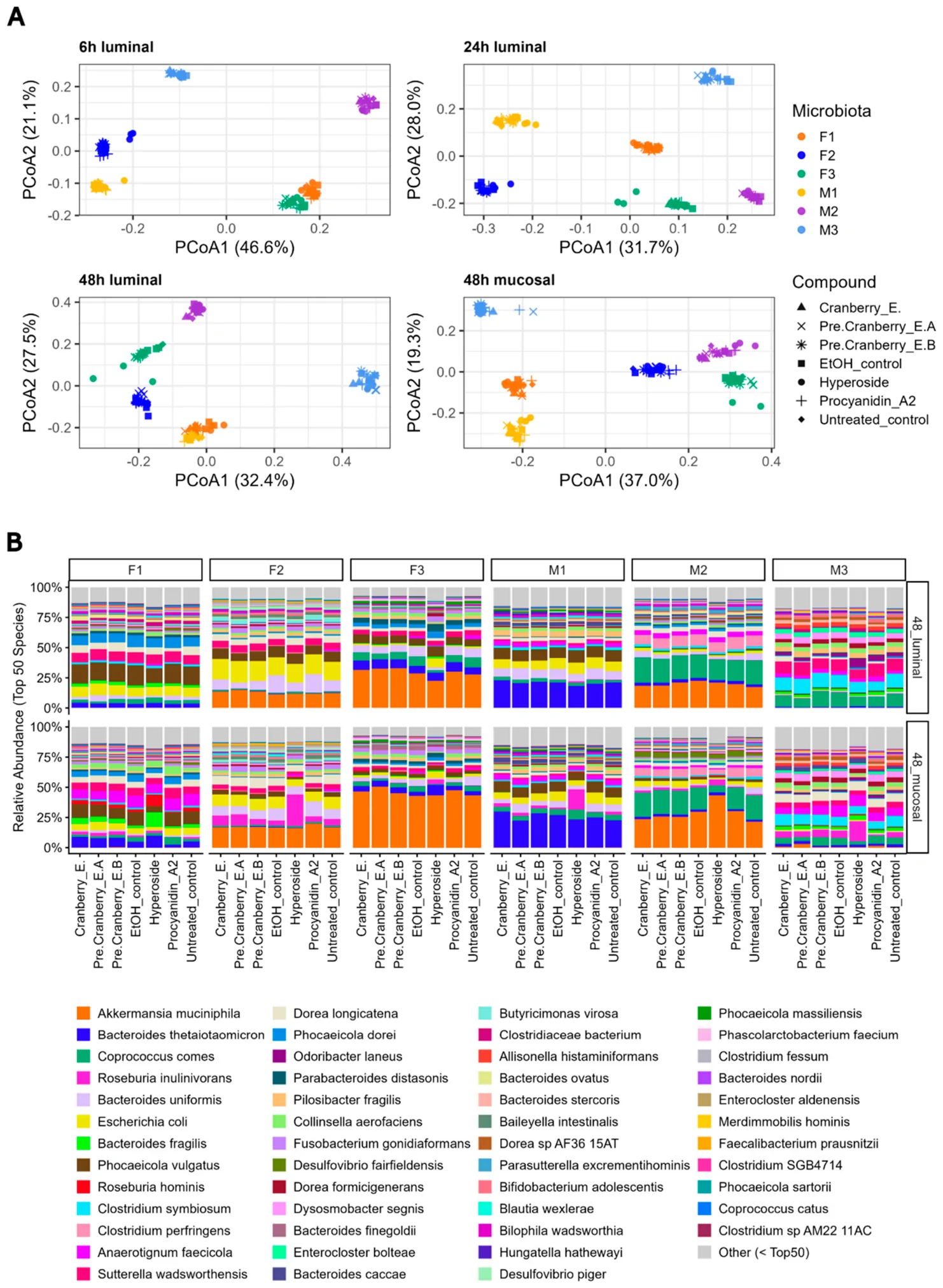
(**A**) Principal coordinate analysis (PCoA) of microbiome composition data of the 6 h, 24 h, 48 h luminal and 48 h mucosal microbiota under selected conditions. (**B**) Top 50 taxa of six individual microbiota donors after 48 h incubation under selected conditions. The upper panel shows the results for the luminal microbiome, and the lower panel shows the results for the mucosal microbiome.

**Figure 2:**
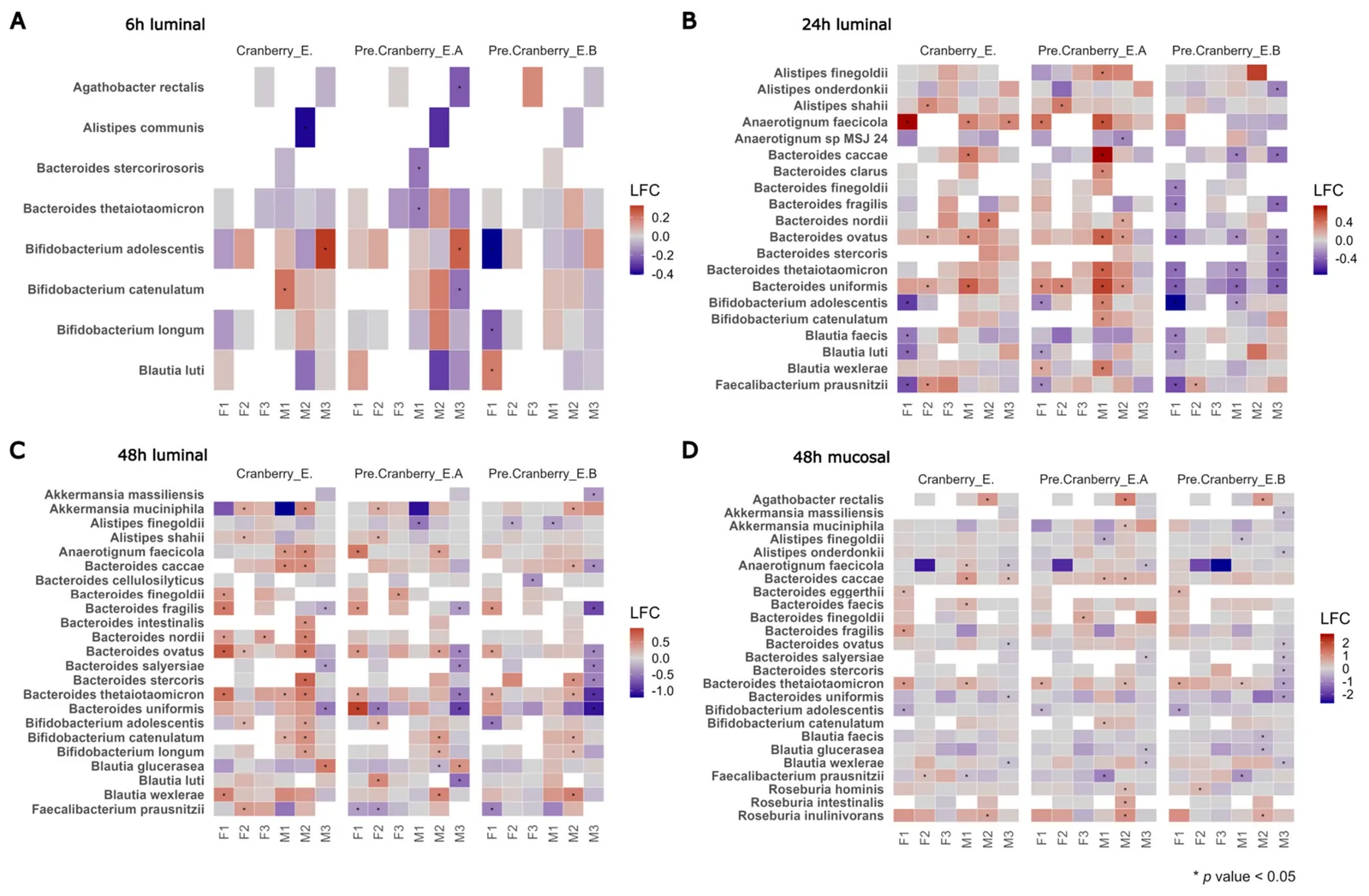
ANCOM-BC analysis showing statistically significant differences between undigested cranberry extract and predigested extracts dissolved in MQ-A and EtOH-B at 6 h (**A**), 24 h (**B**) and 48 h for luminal microbiota (**C**) and 48 h for mucosal microbiota (**D**). The results are shown for a selection of beneficial taxa.

**Figure 3:**
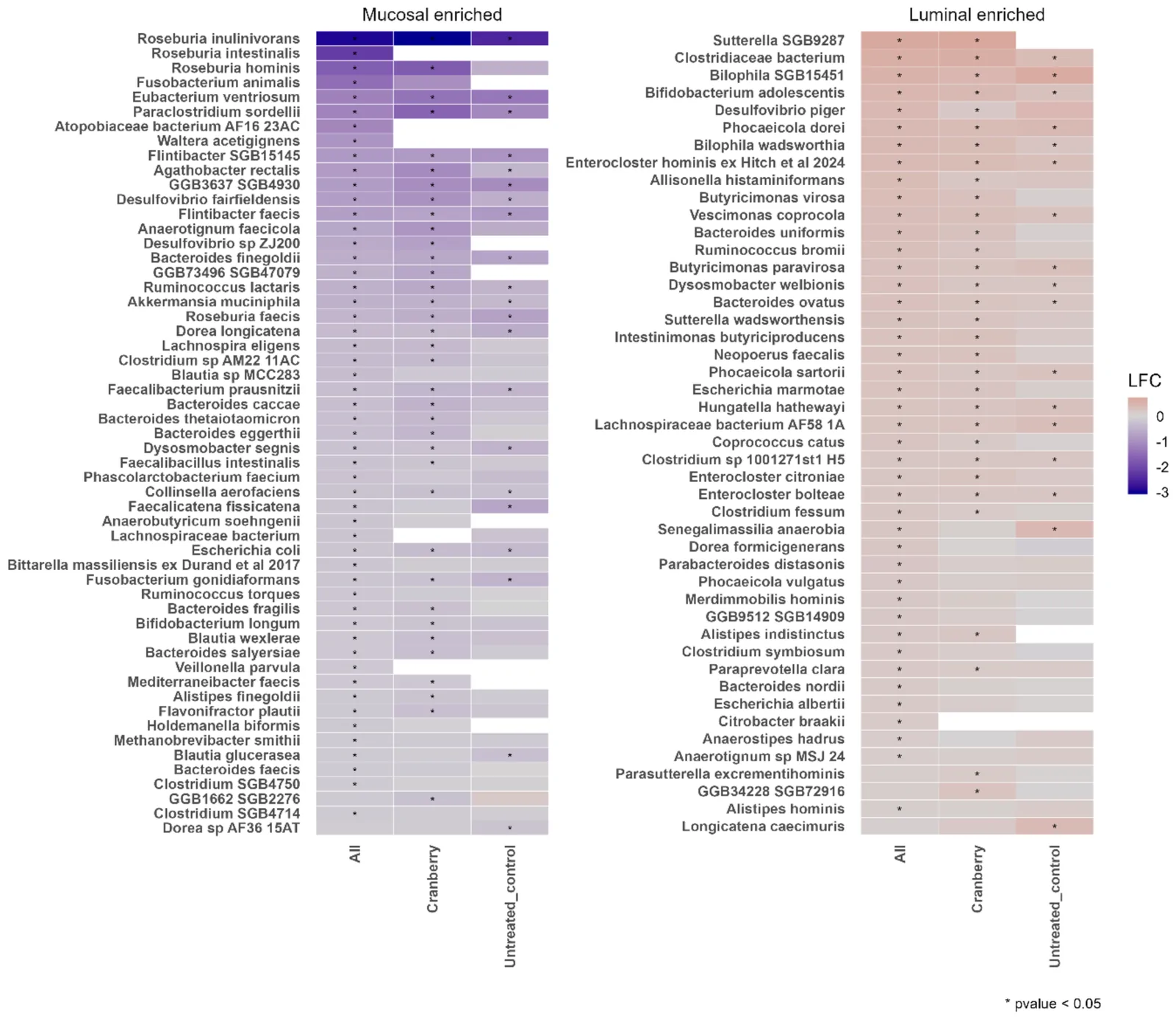
Overview of taxa enriched in the mucosal samples (left panel) or in the luminal samples (right panel) after 48 h exposure. Taxa that were significantly enriched are indicated with an asterisk (*p* value < 0.05). The results are shown for all intervention conditions combined, for cranberry extract exposure only and for the untreated control only.

**Figure 4:**
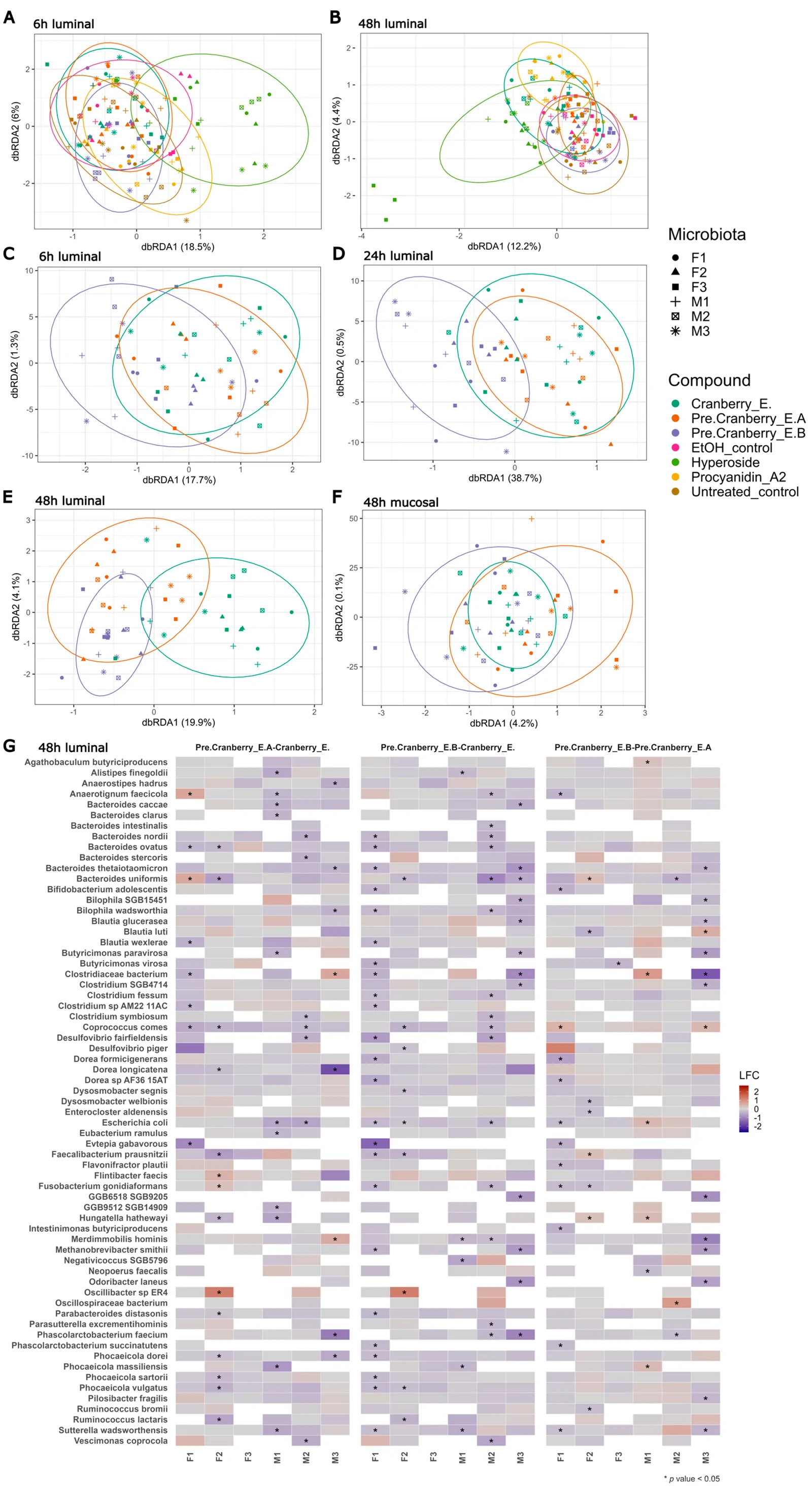
dbRDA analysis modeling the linear relationships at 6 h (**A**) and 48 h (**B**) of luminal microbiota, including controls, corrected for individual variations. dbRDA analysis modeling the linear relationships between the data for the 6 h, 24 h, and 48 h luminal and 48 h mucosal (**C**–**F**) microbiota for cranberry extract conditions corrected for individual variations. The ANCOM-BC analysis showing statistically significant differences between undigested cranberry extract and predigested extracts dissolved in MQ (E.A.) and EtOH (E.B.) at the timepoints indicated in the figure. The results are presented as the log fold change (LFC) and conditions with a *p* value < 0.05 are marked with an asterisk (**G**).

**Figure 5:**
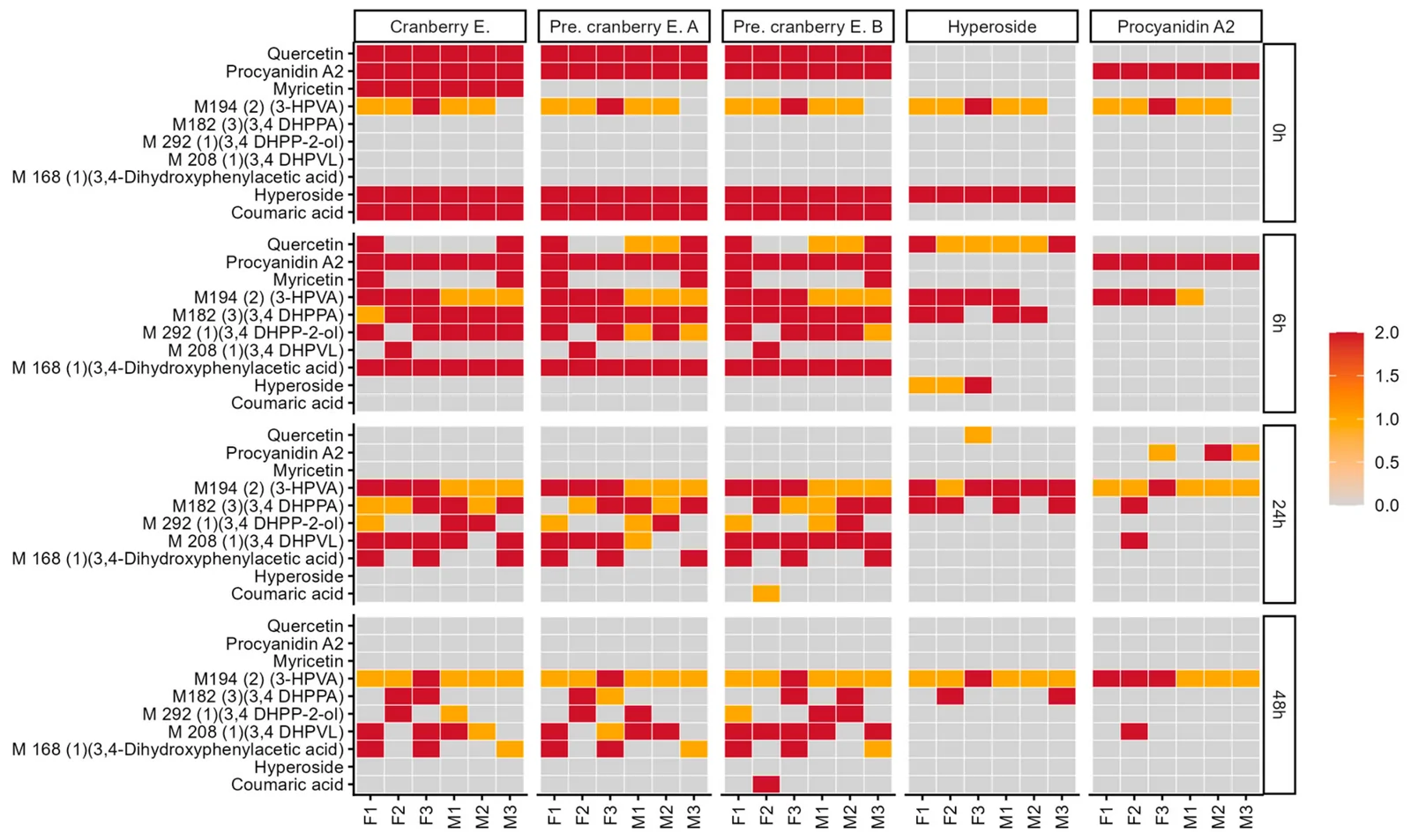
Results from LC-MS analysis of PP metabolites at t = 0 h, 6 h, 24 h, and 48 h. Results are presented on a semiquantitative scale.

**Figure 6:**
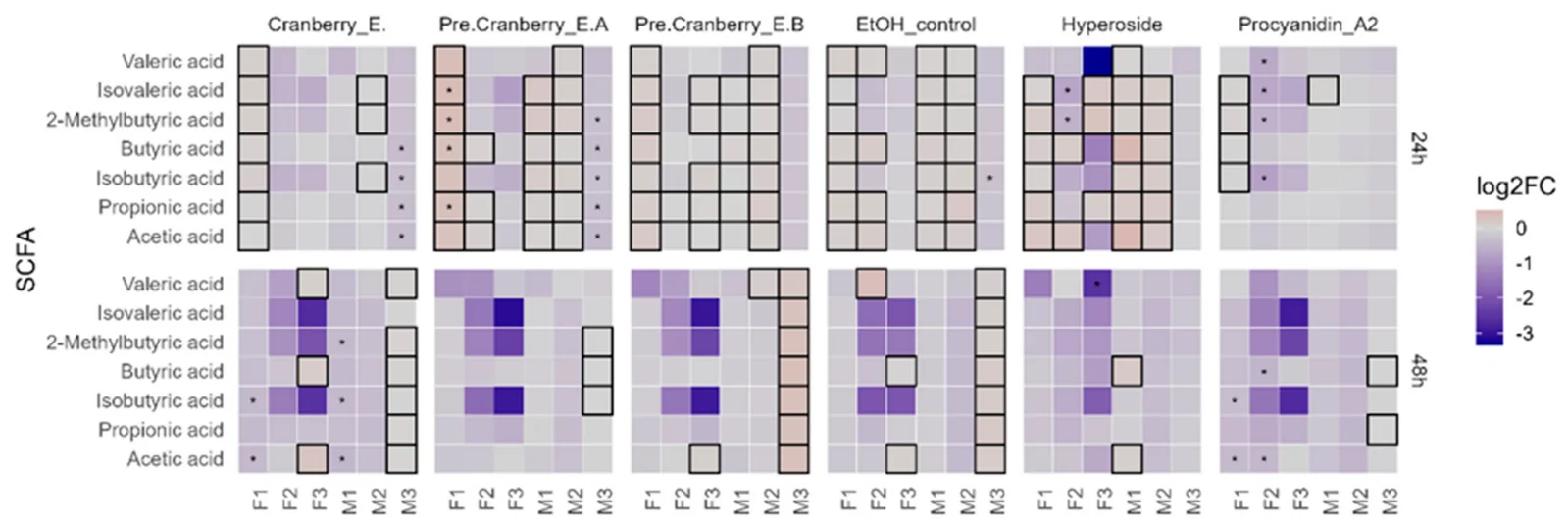
SCFA production shown as the log2 fold change compared to the untreated control. Black-lined boxes indicate conditions with a log2FC > 0. Conditions with a *p* value < 0.05 are marked with an asterisk. The upper panels show the results after 24 h, while the lower panels show the results after 48 h.

**Figure 7:**
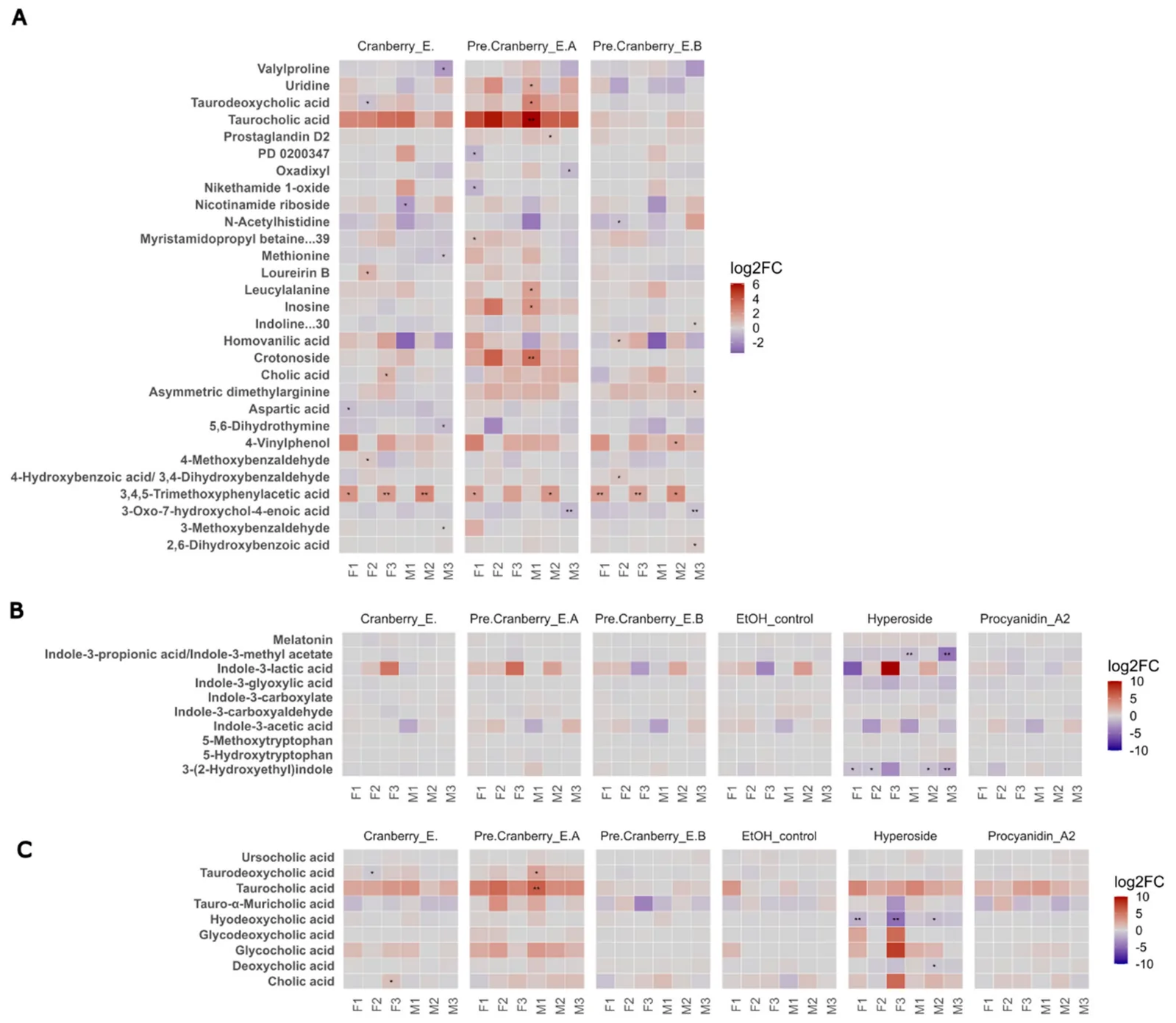
ANCOM-BC analysis showing semipolar compounds that were significantly different from the untreated control sample. Panel (**A**) shows all compounds, revealing significant effects of the three cranberry extracts. Panel (**B**) shows the results for a targeted selection of indoles and tryptamines, while Panel (**C**) shows the results for bile acid metabolites. * p value < 0.05; ** *p* value < 0.01.

**Figure 8:**
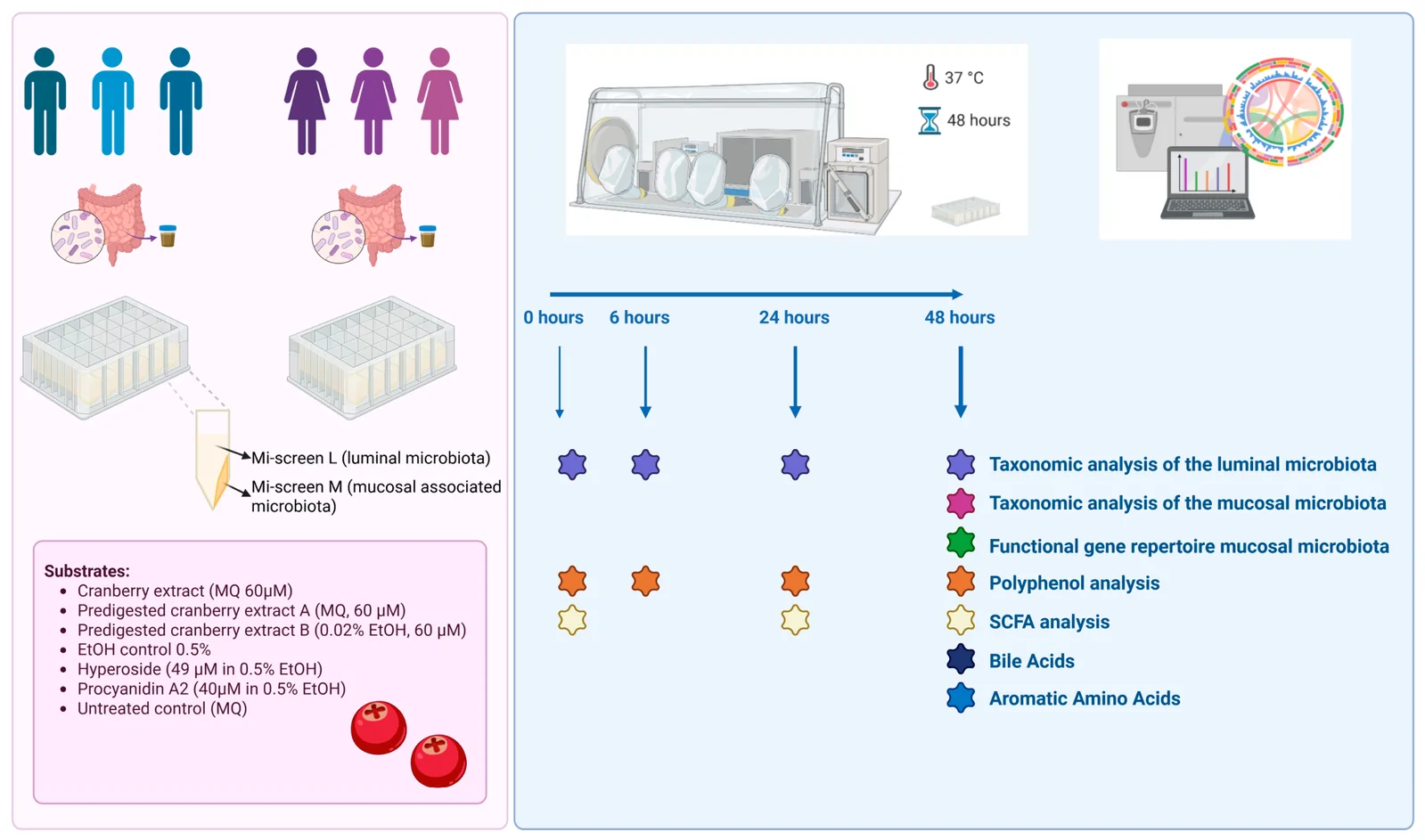
Overview of the experimental setup used for the in vitro screening. Polyphenolic test substrates were screened with six gut microbial communities derived from three male and three female donors. A 24-deepwell plate-based set-up including a mucus agar layer was used, namely the Mi-screen, to test the effects of selected polyphenolic test substrates on the luminal- and mucosa-associated microbiota in vitro. Selected samples were taken throughout the incubation time to evaluate the metabolization of substrates and their effects on the microbial communities and metabolites. Created with BioRender.com.

## Data Availability

The sequencing data were deposited in the ENA database under accession number PRJEB123876.

## Supplementary Materials

The following supporting information can be downloaded at: https://www.mdpi.com/article/10.3390/ijms27198722/s1.
